# Impact of the COVID-19 Lockdown on Ophthalmological Assistance in the Emergency Department at a Spanish Primary Level Hospital

**DOI:** 10.1155/2021/8023361

**Published:** 2021-11-23

**Authors:** Julio González-Martín-Moro, Elena Guzmán-Almagro, Carlos Izquierdo Rodríguez, Ana Fernández Hortelano, Inmaculada Lozano Escobar, Fernando Gómez Sanz, Inés Contreras

**Affiliations:** ^1^Department of Ophthalmology, Hospital Universitario del Henares, Madrid, Spain; ^2^Department of Health Sciences, Universidad Francisco de Vitoria, Madrid, Spain; ^3^Faculty of Optics and Optometry, Universidad Complutense de Madrid, Madrid, Spain; ^4^Department of Ophthalmology, University Hospital Ramón y Cajal, Madrid, Spain; ^5^Clínica Rementería, Madrid, Spain

## Abstract

**Purpose:**

To analyze the changes in ophthalmological emergencies during the COVID-19 pandemic lockdown at a Spanish primary level hospital.

**Methods:**

The number and type of emergencies attended in the emergency department of Hospital Universitario del Henares between March 10 and August 31, 2020 (COVID-19 cohort) were compared with the emergencies attended during the same period of 2019 (pre-COVID-19 cohort). Data on the diagnosis, patient age, and gender was retrospectively collected from the electronic medical records of the hospital. The different diagnoses were organized into “clusters,” which include those conditions that affect the same ocular tissue and that have similar clinical expression.

**Results:**

The number of ophthalmological emergencies during the study period was 841, compared to 1343 during the same month of 2019, which represents a reduction of 37.4%. The percentage reduction in each cluster was as follows: conjunctiva (−65.4%), cornea (−35.8%), uveitis (−3.6%), eyelid and orbital and lacrimal (−35.5%), strabismus (−60%), neuro-ophthalmology (−11.8%), retina (−10.6%), cataract (+16.4%), glaucoma (−37%), and miscellaneous (−45.1%). The number of people seen with viral conjunctivitis decreased by −87.1% compared to 2019. Patients with complications due to conjunctivitis also decreased: patients with pseudomembranes dropped from 16 to 4 cases and patients with corneal subepithelial infiltrates from 9 to 3 cases.

**Conclusions:**

Most diagnostic clusters showed a similar decrease. Clusters that included vision-threating conditions (retina, neuro-ophthalmology, and uveitis) remained mostly stable. During the COVID-19 lockdown, the diagnosis of adenoviral conjunctivitis decreased nearly 10 times. This fact may represent a decrease in the transmission of these infections.

## 1. Introduction

During 2020, COVID-19 (coronavirus disease 2019) completely transformed our world. The impact of the pandemic and the social and behavioral changes implemented to fight it have constituted an unprecedented social experiment. Few articles have reported on the changes in the epidemiology of ocular conditions due to COVID-19.

These changes can be explained by at least five factors. First, SARS-CoV2 can be directly involved as an etiological agent in some inflammatory or vascular diseases, such as conjunctivitis, episcleritis, papillophlebitis, or oculomotor palsies [1–8]. Second, the measures implemented to control the pandemic can also produce changes in the epidemiology of other diseases. For example, social distancing and hygienic measures may have reduced the incidence of some infectious diseases [9, 10]. Third, psychological mechanisms may have increased the incidence of diseases related to stress, such as central serous retinopathy and ocular herpes disease. Fourth, the lockdown and the fear of infection while visiting the hospital may also have influenced the prognosis of some diseases by delaying the necessary medical attention [11–14]. Indeed, an increase in the number of macula-off retinal detachments has been reported, attributed to the delay in seeking ophthalmological care [15]. Finally, a context in which healthcare systems have been overwhelmed may have induced more responsible use of the healthcare resources by citizens [16, 17].

The aim of this study was to evaluate the impact of COVID-19 and anti-COVID-19 measures on the epidemiology of ocular conditions attended in the ophthalmological emergency department of a primary hospital. Particular attention was paid to changes in the epidemiology of infectious conjunctivitis, as some authors have previously reported that anti-COVID-19 measures may have reduced the incidence of this condition [10].

## 2. Materials and Methods

This study was designed as a retrospective cohort study and was approved by the Research Committee of the Hospital Universitario del Henares. The study compared patients who attended the emergency department of this hospital during two time periods. The Hospital Universitario del Henares is a primary level hospital with a catchment area of 175,000 inhabitants in Madrid, Spain, attending over 3500 ophthalmological emergencies every year.

We defined two cohorts of patients. Patients who were attended in the emergency department between March 11, 2020, and August 31, 2020, constituted the post-COVID-19 cohort. These patients were compared with the pre-COVID-19 group. This “control cohort” included patients who attended between March 11, 2019, and August 31, 2019.

Demographic data included age, date of consultation, and gender. Clinical data consisted of the affected eye and the clinical impression, codified into 108 possible diagnoses that were organized into 14 diagnostic clusters. These clusters include those conditions that affect the same ocular tissue and that have similar clinical expression, namely, cornea, conjunctiva, sclera, eyelid, orbit, lacrimal system, glaucoma, crystalline lens, uveitis, strabismus, retina, neuro-ophthalmology, refractive error, and miscellaneous. Those visits in which clinical examination was normal and no diagnosis was made were labelled as “normal ocular examination.” Data were collected on an Excel sheet and analyzed using the SPSS software program (IBM SPSS Statistics for Windows, version 22 (IBM Corp., Armonk, N.Y., USA)). In order to facilitate data management and analysis, some of the less prevalent conditions were grouped with similar or related diagnosis so that the initial 108 diagnoses were recoded into 75 conditions. The number of patients with each diagnosis in each period was compared using tables. The change in cumulative incidence (CI) was expressed as the change in incidence from the previous year (this figure is negative in case of decreasing and positive in case of increasing). The electronic medical records of patients diagnosed with conjunctivitis were reviewed, and the number of patients suffering from corneal subepithelial infiltrates (CSEI) or membranes/pseudomembranes was recorded.

The study was approved by the Research Committee of University Hospital of Henares and followed the principles of the Declaration of Helsinki.

## 3. Results

**Figure 1:**
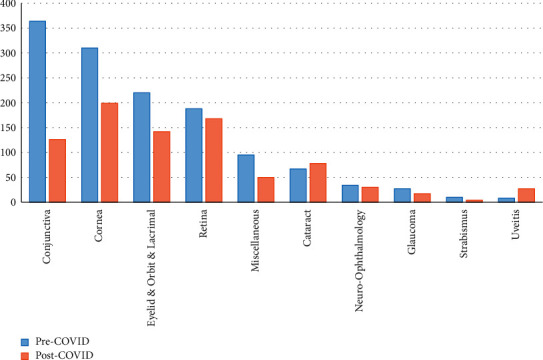
Cluster distribution of patients attended at the emergency department during the pre-COVID-19 and post-COVID-19 period.

**Figure 2:**
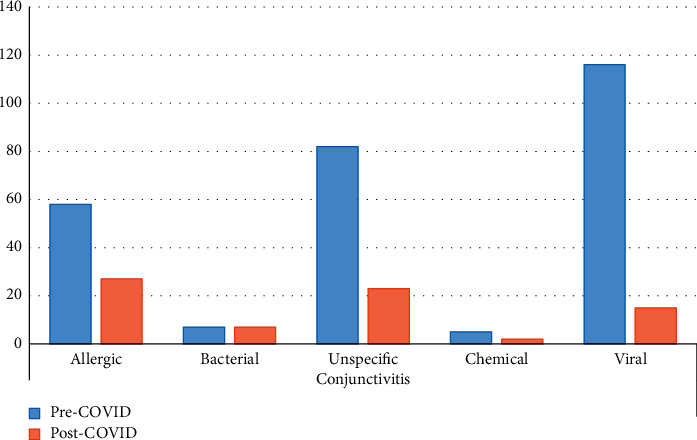
Distribution of patients attended with conjunctivitis at the emergency department during the pre-COVID-19 and post-COVID-19 period.

Demographic data are summarized in Table 1. Mean age was 56.60 years during the pre-COVID-19 period and 56.97 years during the post-COVID-19 one. There was an increase in the proportion of male patients in the post-COVID-19 period (from 43.45% to 47.2%). These changes were not statistically significant (Table 1). The change in the diagnosed conditions and the diagnostic clusters is represented in Figure 1 and detailed in Tables 2 and 3. The number of ophthalmological emergencies attended suffered a 37.3% reduction (841, compared to 1343 during the same months of 2019). The cataract cluster experienced an increase (+16.4%). The remaining clusters suffered reductions. The percentage reduction in each cluster was as follows: conjunctiva (−65.4%), strabismus (−60%), miscellaneous (−45.1%), glaucoma (−37%), cornea (−35.8%), eyelid and orbital and lacrimal (−35.5%), neuro-ophthalmology (−11.8%), retina (−10.6%), and uveitis (−3.6%). The number of people seen with viral conjunctivitis decreased by −87.1% compared to 2019 (Table 2 and Figure 2).

The number of patients with conjunctivitis who developed membranes/pseudomembranes showed an important change from 16 in the 6 months of the pre-COVID-19 period to 4 in the 6 months of the post-COVID-19 period. It is worth noting that three of these complications were seen during the initial days of the pandemic (March and April). During the remaining 4 months (May to August), only one case of conjunctivitis complicated with membranes/pseudomembranes was seen. A similar pattern was observed in the number of patients with conjunctivitis that developed CSEI, which saw a reduction from 9 in the pre-COVID-19 period to 3 in the post-COVID-19 period. Two of these three cases were seen during the first two months of the pandemic.

The proportion of macula-off retinal detachments was 80% (8 out of 10) during the pre-COVID-19 period and 75% (12 out of 16) during the post-COVID-19 period (Table 2), and the difference in median final visual acuity of the retinal detachments operated during both periods was not statistically significant (0.2 during the pre-COVID-19 period and 0.3 during the post-COVID-19 period).

Lastly, a transversal analysis grouping visits related to previous surgery showed a 2.5-fold increase, while the grouping of traumatic conditions decreased to half the cumulative incidence of the previous year.

## 4. Discussion

Data on the impact of COVID-19 and the lockdown on eye care is scarce [18], but ophthalmology is believed to be one of the specialties which was most impacted, with an estimated decrease of 97% in cataract surgery during the initial weeks of the pandemic [18]. As other authors have suggested, the pandemic seems to have led to a more responsible use of emergency medical services in our center. This is supported by significant decreases in the cumulative incidence of mild inflammatory pathologies such as dry eye, conjunctivitis, or blepharitis, while pathologies causing the loss of vision such as retinal detachments or vascular occlusions remained stable (Figure 1, Tables 1, 2). On the other hand, reduced access to sight-saving treatment due to the lockdown and fear of infection may result in an increase in the rates of blindness and ocular disability in the coming years [18]. The interaction between COVID-19 and ocular emergencies is a highly complex phenomenon. As noted, COVID-19 may have altered the incidence of urgent conditions at an emergency eye care department through at least the five mechanisms mentioned above.

During the COVID-19 period, we did not observe a significant change in the mean age of ophthalmic emergencies. Although an increase in the incidence among males was observed, the difference was not statistically significant. Both the stable age and the increase in the proportion of males found in this study are in line with the findings of the study by Poyser et al. using a larger sample from a tertiary English hospital during a shorter time (one month) [9] (Table 1).

The four most common diagnoses at the emergency department during the pre-COVID-19 period were viral conjunctivitis (116 cases), PVD (97 cases), stye/chalazion (63 cases), and blepharitis (57 cases) (Table 2). The most striking finding was the decrease in cases of adenoviral conjunctivitis, which fell from 116 to 15 cases, a drop to only 13% of the cumulative incidence during the control year. No other major diagnoses showed such a dramatic decline (Tables 2 and 3).

This decrease in the number of cases of conjunctivitis has also been reported by other authors [10], who suggested that measures taken to control COVID-19 may have limited the spread of infectious conjunctivitis. However, they did not differentiate among etiologies [10]. Although there are many factors which may influence the decision of a patient with red eye to seek medical care, we believe that a real reduction in the incidence of viral conjunctivitis explains this change. Other nonsevere conditions, such as blepharitis, bacterial conjunctivitis, or chalazion, also showed a decline, but the decrease was smaller (Figure 2Table 2). We believe social and behavioral changes caused by the fight against COVID-19 interrupted adenoviral transmission. Indeed, bacterial conjunctivitis, which in many cases has less severe manifestations, remained stable, perhaps due to the fact that these are in many cases caused by saprophytic flora.

Adenoviral conjunctivitis may be a good indicator of how social changes due to COVID-19 pandemic impact the incidence of other transmissible diseases. There has been a great deal of concern about the coexistence/coinfection of COVID-19 and the flu during the coming waves, and the declines seen in the incidence of adenoviral conjunctivitis make us optimistic regarding this scenario.

The potential of an infectious agent to produce an epidemic is measured using a summary parameter called the basic reproduction number (*R*_0_). It specifies the average number of secondary infections caused by one infected individual during their entire infectious period at the start of an outbreak. *R*_0_ is used to assess the severity of the outbreak, as well as the degree of medical and/or behavioral intervention necessary for control [19]. Adenoviral conjunctivitis has an estimated *R*_0_ of approximately 3 [20], similar to SARS-CoV-2 *R*_0_. If the use of facial masks, social distancing, and hand hygiene can reduce the incidence of a disease as contagious as adenoviral conjunctivitis, we expect that these can also reduce the incidence of other infectious diseases with lower *R*_0_, and as a result, the flu, despite an initial fear, was not a major issue this winter. These measures could also result in a significant reduction in the incidence of other infectious diseases such as tuberculosis in the long term.

One important limitation of our study is that the diagnosis of viral conjunctivitis was made on clinical grounds (not confirmed by laboratory testing), given that diagnosis usually takes place in a clinical setting. Thus, a second analysis was performed using membranes/pseudomembranes and CSEI as proxies for viral conjunctivitis. The medical records of all patients with conjunctivitis were reviewed in order to identify the development of these two complications which can serve as robust indicators of adenoviral conjunctivitis. Other infectious agents, such as *Corynebacterium diphtheriae*, *Neisseria gonorrhoeae*, and *β*-hemolytic streptococcus, can cause membranes, while herpes simplex virus and *Chlamydia trachomatis* can cause pseudomembranes [21]. Nevertheless, the incidence of those infections is extremely low in developed countries. Indeed, a recent meta-analysis concluded that universal prophylaxis for ophthalmia neonatorum is probably no longer indicated due to the low incidence of these forms of neonatal conjunctivitis. The reduction of neonatal gonococcal and chlamydial conjunctivitis is obviously the direct consequence of the reduction of those infections among the adult population [22]. In summary, nonadenoviral membranous or pseudomembranous conjunctivitis is rare. PM causes severe discomfort, while CSEI can cause vision loss. Both will drive most patients to seek medical attention. In fact, a recent article on the influence of COVID-19 in ophthalmological emergencies considered both conditions as the cause of undeferrable medical visits [23].

The number of patients developing PM fell dramatically from 16 in the pre-COVID-19 period to 4 in the post-COVID-19 period. In three cases, the removal of pseudomembranes took place during the months of March and April, suggesting that these patients could have been infected during the pre-COVID-19 period. A similar reduction in the incidence of CSEI was observed, falling from nine to three cases, two of these similarly seen during the months of March and April. The observed reduction probably reflects the real incidence of this infection because the few cases of these complications (PM and CSEI) were seen during the harder days of the lockdown (March and April). Other authors have demonstrated a similar trend in child infections at the emergency department [24].

The community of Madrid was severely hit by COVID-19 during the first wave, with several thousands of people within our catchment area becoming infected. Our data does not point to SARS-CoV-2 as an important cause of conjunctivitis. As other researchers have reported, COVID-19-associated conjunctivitis appears to be self-limiting, requiring no treatment and without affecting visual acuity or being associated with short-term complications [6]. COVID-associated conjunctivitis is most likely as frequent, mild, and unspecific as that associated with other viral infections such as influenza [25, 26], with very low value for the diagnosis of COVID-19 [27].

Significant changes in the epidemiology of vitreoretinal surgery have been reported during the pandemic. Research by Agarwal et al. found that, during the pandemic, vitreoretinal surgery became the second most common form of surgery after ocular trauma surgery in India [28]. Regarding the vitreoretinal cluster, the incidence of retinal detachment was higher during the COVID-19 period (16 cases vs. 10 cases), which may be due to delayed treatment of retinal breaks. Retinal detachment has an estimated incidence of 10/100,000 cases each year [18]. The incidence during the pre-COVID-19 period was consistent with that figure but increased during the COVID-19 period. Nevertheless, the prognosis of the retinal detachments operated at our hospital was similar to those of the pre-COVID-19 cohort, with a similar proportion of macula-off cases and similar postoperative visual acuities. These results differ from those of Patel et al. who reported delayed presentation, higher proportion of macula-off cases, and worse prognosis among COVID-19 retinal detachments during the peak of the pandemic [29], but similar to the findings of Akram et al. and Kaupke et al. who found no differences in the distribution of macula-on and macula-off retinal detachments before and after the pandemic [30, 31].

SARS-CoV-2 is known to be a thrombotic virus [32, 33], and ictus and other ischemic events have been reported as potential manifestations of infection. Although some isolated cases of retinal venous thrombosis have been reported [34–37], our study found that the cumulative incidence of central venous occlusion and branch retinal venous occlusion remained stable. Most likely, retinal vessels are not as vulnerable to COVID-19-induced thrombosis as other vessels, and these complications are more commonly seen in patients hospitalized with severe COVID-19 infection. Similarly, no increase was found in the incidence of oculomotor palsies or ischemic optic neuropathies.

Some studies have reported an increase in traumatic pathologies related to more home improvement activities being performed during the lockdown [38]. However, in our sample, the incidence of traumatic pathologies declined, perhaps due to the restrictions on professional activities. Conversely, an increase in the number of visits related to previous surgery was observed during the pre-COVID-19 period. This increase is probably related to the suspension of postsurgical scheduled visits.

Other studies have analyzed the impact of lockdown on ophthalmologic emergencies [39]. However, to the best of our knowledge, this is the study carried out with greater diagnostic detail and one of the studies that has analyzed a longer period. The choice of a primary care hospital implies both strengths and limitations. Urgent medical attention is only provided in our hospital during working days, from 8.00 to 15.00. Outside these hours, patients must seek medical attention in other hospitals, although they are usually referred back the next day for follow-up at the emergency department. The fact that our hospital does not provide continuous ocular emergency attention may be a source of bias. Nevertheless, this bias should have had a similar impact during both studied periods. Another source of bias is the small number of patients in certain clusters. Glaucoma and strabismus cluster included a small number of patients, making difficult to obtain solid conclusions from the analysis of these groups.

On the other hand, the primary nature of the care provided and the location of the hospital make it easily accessible to citizens. Most of the published studies have been carried out in referral hospitals and are more exposed to selection bias. We thus believe our data are less contaminated by any selection bias than that from tertiary centers and provide a better indication of the true characteristics of urgent ocular pathology. One of the main conclusions of our study is that COVID-19 may have had a significant impact in the incidence of conjunctivitis, something that may be difficult to ascertain using data from a referral center.

We can therefore conclude that COVID-19 and anti-COVID-19 measures effectively reduced the number of patients who attended the hospital emergency department during the pandemic. Clusters which include vision-threating conditions (retina, neuro-ophthalmology, and uveitis) experienced a smaller decrease. Anti-COVID-19 measures appear to have had a positive impact on the transmission of adenoviral conjunctivitis, virtually eradicating this disease during the studied period.

## Figures and Tables

**Table 1:** Demographic distribution of the studied sample.

	Cohort		
	Pre-COVID-19 (*n* = 1343)	Post-COVID-19 (*n* = 841)	*P* value
Age	56.60 (SD = 19.1)	56.97 (SD = 19.2)	0.659^(1)^
Gender distribution (M/F)	M = 583 (43.4%) F = 760 (56.6%)	M = 398 (47.3%) F = 443 (52.7%)	0.077^(2)^

M = male. F = female. Contrasts: (1) Student's-*t*; (2) Chi-square.

**Table 2:** Diagnosis distribution of the most prevalent clusters.

Cluster	Primary diagnosis	Secondary diagnosis	Pre-COVID-19	Post-COVID-19	Change (%)
Conjunctiva	Conjunctivitis	Allergic	58	27	−53.4
		Bacterial	7	7	0
		Unspecific	82	23	−72
		Chemical	5	2	−60
		Viral	116	15	−87.1
		Total	268	74	−72.4
	Subconjunctival bleeding		58	20	−65.5
	Conjunctival lymphangiectasia		9	4	−55.6
	Pterygium/pinguecula		12	8	+33.3
	Conjunctival traumatism		8	9	+12.5
	Others		9	11	+22.2
		Total	96	52	−45.8
	Total conjunctiva		364	126	−65.4

Cornea and sclera	Corneal ulcers and keratitis	Recurrent corneal erosion	3	8	+167.7
		Actinic keratitis	5	1	−80
		Acanthamoeba keratitis	1	0	−100
		Bacterial keratitis	13	3	−76.9
		Herpetic/metaherpetic keratitis	18	25	+38.9
		Contact lens keratitis	13	7	−46.2
		Chemical keratitis	10	9	−10
		Traumatic keratitis	37	26	−29.7
		Other ulcers and keratitis	10	14	+40
	Foreign body	Corneal metallic foreign body	36	28	−22.2
		Other foreign body	18	6	−66.7
	Dry eye and dry eye related keratitis		98	48	−51
	Corneal subepithelial infiltrates (CSEI)		4	4	0
	Sclera	Episcleritis	19	10	−47.4
	Others		25	10	−60
	Total cornea and sclera		310	199	−35.8

Eyelid and orbit and lacrimal	Orbit	Preseptal cellulitis	4	4	0
		Orbit traumatism	6	1	−83.3
		Others	3	2	−33.3
	Inflammatory palpebral disorders	Blepharitis	57	35	−38.6
		Stye/chalazion	63	34	−46
		Unspecific eyelid inflammation	8	8	0
		Palpebral herpes	5	11	+120
	Eyelid malposition	Trichiasis/distichiasis	22	20	−9.1
		Ectropion	2	1	−50
		Entropion	4	1	−75
		Floppy eyelid	1	1	0
		Facial palsy	1	1	0
		Tarsal foreign body	8	6	−25
		Others	19	7	−63.2
	Lacrimal disorders	Acute dacryocystitis	8	5	−37.5
		Others	9	5	−44.4
	Total eyelid and orbit and lacrimal		220	142	−35.5

Retina	Rhegmatogenous retinal disorders	PVD	97	79	−18.6
		Photopsias (isolated)	3	2	−33.3
		Rhegmatogenous lesion	9	8	−11.1
		Retinal rhegmatogenous detachment	10 (8/10 macula-off)	16 (12/16 macula off)	+60
	AMD	AMD (dry)	2	1	−50
		AMD (wet)	3	11	+266.7
	CSC		3	2	−33.3
	Other macular neovascularizations		3	6	+100
	Diabetic retinopathy	Diabetic ME	4	4	0
		Diabetic vitreous hemorrhage	6	5	−16.7
	Vascular occlusions	CRAO/BRAO	1	0	−100
		CRVO/BRVO	10	10	0
	Others		37	24	−35.1
	Total retina		188	168	−10.6

PVD = posterior vitreous detachment; AMD = age related macular degeneration; CSC = central serous choroidopathy; CRAO = central retina artery occlusion; BRAO = branch retina artery occlusion; CRVO = central retina vein occlusion; BRVO = branch retina vein occlusion.

**Table 3:** Diagnosis distribution of the less prevalent clusters.

Cluster	Primary diagnosis	Secondary diagnosis	Pre-COVID-19	Post-COVID-19	Change (%)
Cataract	Cataract		14	19	+35.7
	Posterior capsule opacification		33	48	+45.5
	Cataract surgery complications (other)	Postsurgical macular cystoid edema	2	2	0
		Postsurgical endophthalmitis	0	1	NC
		Rebound uveitis	12	4	−66.7
		Other surgical-related visits	6	4	−33.3
	Total		67	78	+16.4
Glaucoma	Glaucoma drugs side effects		14	5	−64.3
	High IOP detected in other settings		4	4	0
	Glaucoma surgery-related visit		2	5	+150
	Acute angle closure glaucoma		2	0	−100
	Others		5	3	−40
	Total		27	17	−37
Neuro-ophthalmology	Migraine and other headaches		18	18	0
	Optic neuropathies	NAOIN	3	1	−66.7
		Other optic neuropathies	0	2	NC
	Visual pathway lesions		2	0	−100
	Nonfiliated visual loss, ocular pain, and others		11	9	−18.2
	Total neurophthalmology		34	30	−11.8
Strabismus	Strabismus	Oculomotor palsy	8	3	−62.5
		Other strabismus	2	1	−50
	Total strabismus		10	4	−60
Uvetis	Uveitis	Anterior acute uveitis	21	21	0
		Intermediate uveitis	1	2	+100
		Posterior uveitis	0	2	NC
		Traumatic uveitis	5	2	−60
		Others	1	0	−100
		Total	8	27	−3.6
Miscellaneous	Nonpathological ocular examination		61	33	−45.9
	Refractive error		8	9	+12.5
	Leave without examination		14	6	−57.1
	Others		12	2	−83.3
			95	50	−47.4

NAION = nonarteritic optic ischemic neuropathy; NC = not calculable.

## Data Availability

Data are available upon reasonable request.
